# Adverse maternal outcomes and associated factors among mothers of advanced age delivering at a tertiary hospital, southwestern Uganda: a cross-sectional study

**DOI:** 10.1186/s12884-024-06557-1

**Published:** 2024-05-07

**Authors:** Sezalio Masembe, Richard Migisha, Godwin Turyasingura, Hillary Aheisibwe, Emmanuel Nzabandora, John C. Lule

**Affiliations:** 1 Department of Obstetrics and Gynaecology, Kabale School of Medicine, Kabale University, P.0 Box 317, Kabale, Uganda; 2 Department of Physiology, Mbarara University of Science and Technology, Mbarara, Uganda; 3 Department of Obstetrics and Gynaecology, Kabale Regional Referral Hospital, Kabale, Uganda

**Keywords:** Advanced maternal age, Adverse maternal outcomes, Delivery outcomes, Maternal mortality, Uganda

## Abstract

**Background:**

Mothers of advanced age, defined as pregnant women aged ≥ 35 years at the time of giving birth, are traditionally known to be associated with increased risks of adverse maternal outcomes. We determined the prevalence of adverse maternal outcomes and associated factors among mothers of advanced age who delivered at Kabale Regional Referral Hospital (KRRH), in Southwestern Uganda.

**Methods:**

We conducted a cross-sectional study at the Maternity Ward of KRRH from April to September 2023. We consecutively enrolled pregnant women aged ≥ 35 years during their immediate post-delivery period and before discharge. We obtained data on their socio-demographic, obstetric, medical characteristics and their maternal outcomes using interviewer-administered questionnaires. We defined adverse maternal outcome as any complication sustained by the mother that was related to pregnancy, delivery and immediate post-partum events (obstructed labour, antepartum haemorrhage, mode of delivery [cesarean or vacuum extraction], postpartum haemorrhage, hypertensive disorders of pregnancy, preterm or postdate pregnancy, anemia, premature rupture of membranes, multiple pregnancy, and maternal death). A participant was considered to have an adverse outcome if they experienced any one of these complications. We identified factors associated with adverse outcomes using modified Poisson regression.

**Results:**

Out of 417 participants, most were aged 35–37 years (*n* = 206; 49.4%), and had parity ≥ 5 (65.5%). The prevalence of adverse maternal outcomes was 37.6% (*n* = 157, 95%CI: 33.1–42.4%). Common adverse maternal outcomes included caesarian delivery (23%), and obstructed labour (14.4%). Other complications included anemia in pregnancy (4.5%), chorioamnionitis (4.1%), preterm prelabour rupture of membranes (3.9%), and chronic hypertension and preeclampsia (both 2.4%). Factors associated with adverse maternal outcomes were precipitate labour (adjusted prevalence ratio [aPR] = 1.95, 95%CI: 1.44–2.65), prolonged labour, lasting > 12 h (aPR = 2.86, 95%CI: 1.48–3.16), and chronic hypertension (aPR = 2.01, 95%CI: 1.34–3.9).

**Conclusion:**

Approximately two-fifth of the advanced-aged mothers surveyed had adverse outcomes. Mothers with prolonged labour, precipitate labour and chronic hypertension were more likely to experience adverse outcomes. We recommend implementation of targeted interventions, emphasizing proper management of labor as well as close monitoring of hypertensive mothers, and those with precipitate or prolonged labor, to mitigate risks of adverse outcomes within this study population.

## Introduction

Advanced aged mothers refer to those mothers aged 35 years or older during pregnancy or childbirth [1]. Globally, there is an observable trend of an increasing average age at childbearing [2, 3]. This shift is facilitated by the widespread adoption of assisted reproductive technology, extending to menopausal women through egg donor programs. Contributing factors to this trend include intentional delays due to career commitments, prolonged professional paths, postponed marriages, and the continuation of childbearing into later stages of life [4].

Pregnancies among advanced aged mothers are known to be associated with adverse maternal outcomes including obstructed labour, antepartum haemorrhage, postpartum haemorrhage, operative delivery, hypertensive disorders of pregnancy, [5–7]. Other adverse outcomes include preterm and post-term deliveries, anemia, premature rupture of membranes, multiple pregnancies, and maternal deaths [8–11].

Factors associated with adverse pregnancy outcomes among mothers of advanced age include chronic medical conditions such as diabetes and hypertensive disease, parity, socio-demographic factors such as maternal age, level of education and income and labor duration.[5, 11–13]. Maternal age has been independently associated with adverse outcomes with those beyond 40 years experiencing a higher impact [3, 10, 13].

In Southwestern Uganda, there is a gap in understanding maternal outcomes and associated factors among advanced-aged mothers, as no previous study has addressed this issue. The current study aims to fill this gap by laying the groundwork for evidence-based preventive interventions tailored to mothers in this age group. The findings do not only aim to improve outcomes, but also establish a framework for future research specific to this maternal demographic. Specifically, this study determined the prevalence of adverse maternal outcomes and associated factors among advanced-aged mothers receiving care at a tertiary hospital in southwestern Uganda.

## Methods

### Study setting, study design and study population

We conducted a cross sectional study at the Maternity Ward of Kabale Regional Referral Hospital (KRRH) in Southwestern Uganda. The hospital doubles as a teaching hospital for Kabale University School of Medical as well as a regional referral hospital for districts in southwestern Uganda and neighbouring countries of Democratic Republic of Congo and Rwanda. The Maternity Ward of the hospital conducts approximately 450 deliveries every month, with a caesarean section rate of 18–20%.

We included in our study all mothers aged 35 years and older during their immediate post-delivery period and before discharge who were admitted from March to August 2023.

### Sample size and sampling methods

The sample size(n) was calculated using the Kish-Leslie formula [14] with a 5% significance level and 95% confidence level:

$${\text{n}}=\frac{({{\text{z}}}^{2}{\text{pq}})}{{{\text{d}}}^{2}}$$

where:

n is the total number of participants required.

z is the critical value (it is 1.96 at 0.05 level of significance).

p is the known proportion of pregnant women of advanced age (50% was considered due to lack of local prevalence data).

q is1-p.

d is margin of error (0.05).

*n* = 1.96^2^ × 0.5x0.5/0.05.^2^

*n* = 384.

Accounting for a 10% non-response rate, a total sample size of 427 was obtained as shown below:

$${\text{N}}={\text{n}}/1-{\text{p}}$$

$${\text{N}}=384/0.9$$

$${\text{N}}=427$$

### Data collection and study variables

Data were collected using interviewer administered structured questionnaires after obtaining written informed consent by four trained research assistants. These were midwives closely supervised by the principal investigator. We collected data on independent variables such as maternal socio-demographic, medical and obstetric characteristics and dependent variables were the adverse maternal outcomes. Socio-demographic variables included maternal age, education level, occupation, level of income, marital status and residence. Medical characteristics included pregestational diabetes, chronic hypertension, preexisting heart disease, and long-term medications. Obstetric variables included parity, duration of labour, history of contraceptive use, pregnancy-induced hypertension (eclampsia and pre-eclampsia) and antenatal care visits attendance.

Maternal adverse outcomes were obstructed labour, antepartum haemorrhage, mode of delivery (vacuum extraction or cesarean delivery), postpartum haemorrhage, hypertensive disorders of pregnancy, gestational age at delivery (preterm or postterm), anemia, premature rupture of membranes, multiple pregnancy, and maternal death. We defined adverse maternal outcomes as any of above complications sustained by the mother related to the most recent pregnancy, delivery and immediate post-partum events.

### Data management and analysis

Data was entered in Epi Info software version 7 (CDC, Atlanta, USA) and exported to STATA version 15 (StataCorp, College Texas, USA) for analysis.

Maternal socio-demographic, obstetric, medical characteristics and fetal outcomes were summarized using descriptive statistics and expressed as frequencies/percentages. To determine the prevalence of adverse maternal outcomes, we calculated the percentage of participants experiencing any one of the complications related to pregnancy, delivery, and immediate post-partum events over the total study participants.

For both bivariate and multivariate analyses, we used a generalized linear model regression of the Poisson family with a log link (modified Poisson), with robust standard errors to identify factors associated with adverse outcomes among the study participants. This is because the prevalence of our outcome of interest was high [15, 16]. Variables meeting a significance threshold of *p*-value < 0.2 at bivariate analysis and those with biological plausibility, such as maternal age, were retained in the multivariable regression model. We reported prevalence ratios and their corresponding confidence intervals as measures of association. Significance for all analyses was set at *p* < 0.05.

## Results

**Fig. 1:**
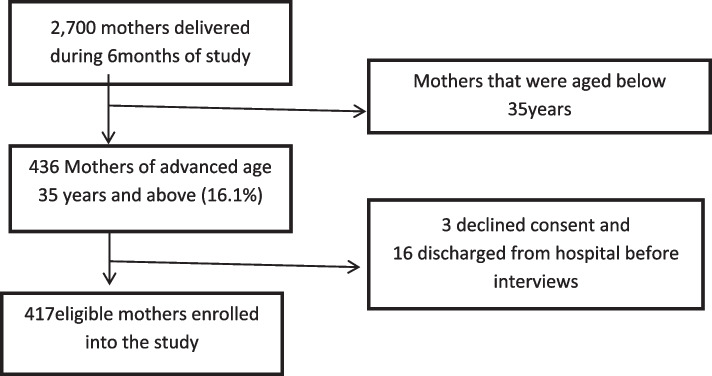
Flow chart for recruitment of study participants

Out of 2,700 mothers who delivered during the study period of six months, 436(16.1%) were of advanced age. Three mothers declined consent to participate while other sixteen went home before completing questionnaires. We enrolled 417 women who were eligible to participate (Fig. 1).

### Demographic, obstetric and medical characteristics of the study participants

Among the 417 participants, most fell within the age range of 35–37 years (49.4%), followed by those aged 38–40 years (36.9%), while only 13.7% were above 40 years old. Educational attainment was predominantly at the primary or secondary level (36.9% and 37.2%, respectively). A significant proportion were married (87.5%), resided in rural areas (65.7%), and were multiparous with five or more previous pregnancies (65.5%). The majority attended at least four antenatal care (ANC) visits (94%) and utilized contraception between pregnancies (77.9%). Chronic illnesses were reported in 3.3% of participants (Table 1).

**Table 1:** Demographic, obstetric and medical characteristics of the participants (*N* = 417)

Characteristic	Frequency	Percent
**Age (years)**		
35–37	206	49.4
38–40	154	36.9
> 40	57	13.7
**Education level**		
No formal education	11	2.6
Primary	154	36.9
Secondary	155	37.2
Tertiary	97	23.3
**Level of income**		
< 1 USD	142	34.1
≥ 1 USD	275	66.0
**Marital status**		
Married	365	87.5
Divorced/single	52	12.5
**Residence**		
Rural	274	65.7
Urban	143	34.3
**Occupation**		
Peasant	205	49.3
Business	128	30.8
Formal employment	45	10.8
Casual labour	38	9.2
**Parity**		
1	26	6.2
2–4	118	28.3
≥ 5	273	65.5
**Duration of active labour**		
< 3 h	6	1.4
3—12 h	346	83.0
> 12 h	65	15.6
**Antenatal care visits**		
< 4	25	6.0
≥ 4	392	94.0
**History of ectopic pregnancy**		
No	414	99.3
Yes	3	0.7
**Contraception use before current pregnancy**		
No	92	22.1
Yes	325	77.9
**Hypertension**		
No	402	96.4
Yes	15	3.6
**Other chronic illnesses**		
No	403	96.7
Yes	14	3.3
**Long-term medications**		
No	373	89.4
Yes	44	10.6

### Prevalence of maternal outcomes

Of the 417 mothers who participated in the study, the majority (62.3%, *n* = 260) did not experience any adverse outcome. The prevalence of adverse maternal outcomes was 37.6% (*n* = 157, 95% CI: 33.1–42.4%) (Table 2).

**Table 2:** Maternal outcomes of study participants (*N* = 417)

Variable	Frequency	Percent
**Any adverse maternal outcome**		
No	269	62.3
Yes	157	37.7
**Type of adverse outcome**^a^		
Obstructed labour	60	14.4
Pre-Term PROM	35	8.4
Postpartum haemorrhage	27	6.5
Anemia in pregnancy	19	4.5
Chorioamnionitis	17	4.1
Multiple pregnancy	13	3.2
Chronic hypertension	10	2.4
Preeclampsia	10	2.4
Gestational diabetes	8	1.9
Term PROM	7	1.7
Antepartum hemorrhage	5	1.5
Eclampsia	4	1.0
**Mode of delivery**		
SVD	317	76.0
Vacuum^a^	4	1.0
Caesarean^a^	96	23.0
Maternal death	1	0.2
**Gestational age at delivery**		
< 37 weeks^a^	5	1.2
37—41 weeks	411	98.6
≥ 42 weeks^a^	1	0.2

*PROM* Prelabour rupture of membranes, *SVD* Spontaneous vaginal delivery, ^a^These adverse outcomes are not mutually exclusive

The most prevalent complication was obstructed labour, affecting 14.4% of participants. Other complications included anemia in pregnancy (4.5%), chorioamnionitis (4.1%), preterm prelabour rupture of membranes (3.9%), and chronic hypertension and preeclampsia (both 2.4%). One maternal death was recorded during the study period. In terms of delivery modes, the majority experienced spontaneous vaginal delivery (76.0%), followed by Caesarean Sect. (23.0%). Four delivered with vacuum assistance. Gestational age at delivery was primarily between 37 and 41 weeks (98.6%), with a small proportion occurring before 37 weeks (1.2%). Multiple pregnancies were identified in 3.1% of participants (Table 2).

## Factors associated with adverse maternal outcomes

Participants who experienced precipitate labor < 3 h (adjusted prevalence ratio [aPR] = 1.95, 95% CI: 1.44–2.65), those that experienced prolonged labor lasting more than 12 h (aPR = 2.86, 95% CI: 1.48–3.16), and chronic hypertension (aPR = 2.01, 95% CI: 1.34–3.9) were more likely to experience adverse outcomes compared to their counterparts (Table 3).

**Table 3:** Factors associated with adverse maternal outcomes among mothers of advanced age at Kabale Regional Referral Hospital, southwestern Uganda

variable	Adverse maternal outcomes							**p value**
	**Yes, (*n* = 157)**	**(%)**	**No, (*n* = 260), n**	**(%)**	**cPR (95%CI)**	**p value**	**aPR (95%CI)**	
**Age category (year)**								
35–37	82	(52.2)	124	(47.7)	Ref		Ref	
38–40	52	(33.1)	102	(39.2)	0.85 (0.64—1.12)	0.246	0.78 (0.60 -1.02)	0.069
> 40	23	(14.7)	34	(13.1)	1.01 (0.71—1.45)	0.941	1.02 (0.65—1.56)	0.921
**Education level**								
No formal education	7	(4.5)	4	(1.5)	2.13 (1.28 – 2.54)	0.004	1.44 (0.78—2.68)	0.244
Primary	46	(29.3)	108	(41.5)	Ref		Ref	
Secondary	58	(36.9)	97	(37.3)	1.25 (0.91—1.72)	0.163	1.13 (0.83—1.52)	0.439
Tertiary	46	(29.3)	51	(19.6)	1.58 (1.15 – 2.19)	0.005	1.20 (0.85—1.70)	0.294
**Level of income**								
< 1 USD	51	(32.5)	91	(35.0)	Ref			
≥ 1 USD	106	(67.5)	169	(65.0)	1.07 (0.82—1.40)	0.602	–	
**Marital status**								
married	21	(13.4)	31	(11.9)	Ref			
divorced/single	136	(86.6)	229	(88.1)	0.92 (0.65 – 1.32)	0.658	–	
**Residence**								
Rural	99	(63.1)	175	(67.3)	Ref			
Urban	58	(36.9)	85	(32.7)	1.12 (0.87—1.45)	0.372	–	
**Occupation**								
Peasant	71	(45.2)	134	(51.7)	Ref			
Business	48	(30.6)	80	(30.9)	1.08 (0.81—1.45)	0.594	–	
Formal employment	22	(14.0)	23	(8.9)	1.41 (0.99 – 2.01)	0.056	–	
Casual labour	12	(7.6)	17	(6.6)	1.19 (0.74 – 1.92)	0.461	–	
**Parity**								
1	12	(7.6)	14	(5.4)	Ref			
2–4	45	(28.7)	73	(28.1)	0.83 (0.51 – 1.33)	0.431	–	
≥ 5	100	(63.7)	173	(66.5)	0.79 (0.51—1.24)	0.308	–	
**Antenatal care visits**								
< 4	14	(8.9)	11	(4.2)	Ref		Ref	
≥ 4	143	(91.1)	249	(95.8)	0.65 (0.45 – 0.94)	0.024	0.76 (0.54—1.08)	0.125
**History of abortions**								
No	110	(70.1)	209	(80.4)	Ref		Ref	
Yes	47	(29.9)	51	(19.6)	1.36(1.08–1.80)	0.012	1.25 (0.96—1.62)	0.091
**Contraception use before current pregnancy**								
No	29	(18.5)	63	(24.2)	Ref		Ref	
Yes	128	(81.5)	197	(75.8)	1.25 (0.90—1.74)	0.186	1.08 (0.79 – 1.48)	0.620
**Duration of active labour**								
< 3 h	5	(3.2)	1	(0.4)	2.05 (1.52 – 2.76)	< 0.001	1.95 (1.44 – 2.63)	< 0.001
3—12 h	104	(66.2)	242	(93.1)	Ref		Ref	
> 12 h	48	(30.6)	17	(6.5)	2.95 (2.30 – 3.80)	< 0.001	2.86 (1.48—3.16)	< 0.001
**Long term medications**								
No	133	(84.7)	240	(92.3)	Ref		Ref	
Yes	24	(15.3)	20	(7.7)	1.53 (1.13—2.07)	0.006	0.98 (0.70—1.37)	0.913
**Diabetes**								
No	150	(95.5)	257	(98.9)	Ref		Ref	
Yes	7	(4.5)	3	(1.2)	1.90 (1.24 – 2.91)	0.003	1.44 (0.90 – 2.30)	0.131
**Hypertension**								
No	144	(91.7)	258	(99.2)	Ref		Ref	
Yes	13	(8.3)	2	(0.8)	2.42 (1.91—3.07)	< 0.001	2.06 (1.34—3.19)	0.001

*aPR* Adjusted prevalence ratio, *cPR* Crude prevalence ratio, *CI* Confidence interval, *Ref* Reference category

## Discussion

In this study, approximately two in five of the advanced aged mothers surveyed experienced at least one adverse maternal outcome. The most common adverse outcomes among these mothers were caesarian delivery and obstructed labour. Advanced aged mothers with prolonged labour, precipitate labour and chronic hypertension were more likely to experience adverse outcomes.

The overall rate of adverse maternal outcomes in the current study was 37.6%. This compares well with study findings from other countries such as 32.1% in Ethiopia [6], 36% in Israel [9, 17], 37.5% in Spain [18] and 36% in the USA[11]. However, the rate of adverse maternal outcomes in the current study was lower than the rates reported in Poland, Brazil, the USA, and Israel, where studies documented a higher proportion of mothers experiencing adverse outcomes ranging from 38 to 56% study [3, 19–21]. This divergence in adverse maternal outcome rates between our study and those in Poland, Brazil, the USA, and Israel can be attributed to the substantial inclusion of participants aged above 40 years in those studies. Advanced maternal age, especially beyond 40 years, is widely recognized as a significant risk factor for adverse outcomes, as corroborated by previous research [3]. Conversely, our study had a limited number of mothers aged 40 years or older, likely contributing to a lower proportion of adverse outcomes observed. Moreover, the comparatively favorable outcomes in our study may be attributed to several protective factors prevalent among our participants; the majority (94%) benefited from adequate antenatal care, minimizing potential complications. Additionally, the low prevalence of chronic medical morbidities among our study population and the widespread use of contraception likely contributed to the observed lower rate of adverse maternal outcomes compared to these studies. Furthermore, a significant proportion of our participants had achieved a fairly high level of education, which is consistently associated with improved maternal health outcomes[22].

The adverse maternal outcomes identified in our study were primarily obstructed labor (14.4%) and operative delivery (23%), alongside less frequent events such as PROM, antepartum hemorrhage, chorioamnionitis, preeclampsia, eclampsia, gestational diabetes, anemia in pregnancy, multiple pregnancies, and postpartum hemorrhage. These patterns in our findings are consistent with observations reported in other studies [2, 6, 9, 10, 13, 23].

In the current study, having prolonged or precipitate labour was associated with adverse outcomes among participants. Abnormal labour duration is consistently associated with maternal morbidities such as operative delivery, postpartum haemorrhage and sepsis [24]. Therefore, we recommend proper labor monitoring among advanced-aged mothers to avoid undue prolongation by intervening timely. In case these mothers experience precipitate labour, close and vigilant monitoring as well as prompt management of associated adverse outcomes should be considered as essential components of comprehensive care for this population.

In this study, having chronic hypertension was also associated with adverse maternal outcomes, consistent with other studies in the USA and the UK [25, 26]. This is because such mothers are faced with a high likelihood of elective operative delivery, gestational diabetes, and preeclampsia or eclampsia. On the basis of this finding, we recommend that advanced aged mothers with chronic hypertension be closely monitored during pregnancy, labour and delivery to avoid and or manage associated adverse outcomes.

This study is subject to certain limitations that merit acknowledgment. One notable constraint arises from the relatively modest sample size within the study group aged 40 years or older; restricting our statistical power to comprehensively assess the influence of very advanced maternal age on adverse perinatal outcomes. Women aged 40–44 years are known to be at more increased risk for adverse outcomes compared with women aged 35–39 years [23]. Additionally, the absence of a cohort of younger mothers for comparative analysis presents a limitation, as the entirety of our study participants were aged 35 years or older. Finally, our study findings are generalizable to advanced-aged women in the Southwestern Uganda region and similar peri-urban sub-Saharan African settings, and may not be generalizable to other study settings or populations. Despite these limitations, the primary strength of our study lies in its pioneering nature as one of the first investigations to explore adverse maternal outcomes among advanced-aged mothers in the East African Region.

## Conclusion

Approximately two in five of the advanced-aged mothers surveyed had adverse outcomes. The most common adverse outcomes among these mothers are caesarian delivery and obstructed labour. Mothers with prolonged labour, precipitate labour and chronic hypertension are more likely to experience adverse outcomes compared to their counterparts. We recommend implementation of targeted interventions, emphasizing proper management of labor, close monitoring of advanced aged mothers, and a deliberate effort to avoid prolonged, to mitigate risks of adverse outcomes within this study population. In case of precipitate labour, such mothers should be closely monitored for adverse outcomes and timely interventions. Mothers conceiving or intending to conceive at advanced age should be counselled about likelihood of the adverse outcomes. Future longitudinal studies are recommended to better understand the immediate and long-term maternal outcomes associated with advanced maternal age in our setting.

## Data Availability

The datasets used and/or analysed during the current study available from the corresponding author on reasonable request.

## Abbreviations

ANC: Antenatal care
FSB: Fresh still birth
IUFD: Intrauterine fetal death
IUGR: Intrauterine growth restriction
LBW: Low birth weight
KRRH: Kabale regional referral hospital
SVD: Spontaneous vertex delivery
WOA: Weeks of amenorrhea
PROM: Premature rupture of membranes
PPH: Post-partum haemorrhage

## References

[1] Pregnancy at Age 35 Years or Older: ACOG Obstetric Care Consensus No. 11. Obstetrics & Gynecology. 2022;140(2):348–66. 10.1097/AOG.0000000000004873 35852294

[2] Laopaiboon M, Lumbiganon P, Intarut N, Mori R, Ganchimeg T, Vogel J (2014). Advanced maternal age and pregnancy outcomes: a multicountry assessment. BJOG: Int J Obstet Gy.

[3] Radoń-Pokracka M, Adrianowicz B, Płonka M, Danił P, Nowak M, Huras H (2019). Evaluation of Pregnancy Outcomes at Advanced Maternal Age. Open Access Maced J Med Sci.

[4] Guedes M, Canavarro MC (2014). Characteristics of Primiparous Women of Advanced Age and Their Partners: A Homogenous or Heterogenous Group?. Birth.

[5] Kenny LC, Lavender T, McNamee R, O’Neill SM, Mills T, Khashan AS (2013). Advanced Maternal Age and Adverse Pregnancy Outcome: Evidence from a Large Contemporary Cohort. Shi Q, editor PLoS ONE.

[6] Mehari M ab, Maeruf H, Robles CC, Woldemariam S, Adhena T, Mulugeta M, et al. Advanced maternal age pregnancy and its adverse obstetrical and perinatal outcomes in Ayder comprehensive specialized hospital, Northern Ethiopia, 2017: a comparative cross-sectional study. BMC Pregnancy Childbirth. 2020;20(1):60. 10.1186/s12884-020-2740-6 PMC6993443 32000714

[7] Mills TA, Lavender T (2011). Advanced maternal age. Obstet Gynaecol Reprod Med.

[8] Almeida NKO, Almeida RMVR, Pedreira CE (2015). Adverse perinatal outcomes for advanced maternal age: a cross-sectional study of Brazilian births. Jornal de Pediatria.

[9] Attali E, Yogev Y (2021). The impact of advanced maternal age on pregnancy outcome. Best Pract Res Clin Obstet Gynaecol.

[10] CakmakCelik F, Aygun C, Kucukoduk S, Bek Y (2017). Maternal and neonatal outcomes in advanced maternal age: a retrospective cohort study. J Matern Fetal Neonatal Med.

[11] Correa-de-Araujo R, Sarah Yoon SS (2021). Clinical Outcomes in High-Risk Pregnancies Due to Advanced Maternal Age. J Women’s Health..

[12] Getaneh T, Asres A, Hiyaru T, Lake S (2021). Adverse perinatal outcomes and its associated factors among adult and advanced maternal age pregnancy in Northwest Ethiopia. Sci Rep.

[13] Ogawa K, Urayama KY, Tanigaki S, Sago H, Sato S, Saito S (2017). Association between very advanced maternal age and adverse pregnancy outcomes: a cross sectional Japanese study. BMC Pregnancy Childbirth.

[14] Kish L. Survey sampling. In: Survey sampling. 2011. p. 643–643.

[15] Barros AJ, Hirakata VN (2003). Alternatives for logistic regression in cross-sectional studies: an empirical comparison of models that directly estimate the prevalence ratio. BMC Med Res Methodol.

[16] Zou G (2004). A Modified Poisson Regression Approach to Prospective Studies with Binary Data. Am J Epidemiol.

[17] Glick I, Kadish E, Rottenstreich M (2021). Management of Pregnancy in Women of Advanced Maternal Age: Improving Outcomes for Mother and Baby. IJWH.

[18] Casteleiro A, Paz-Zulueta M, Parás-Bravo P, Ruiz-Azcona L, Santibañez M (2019). Association between advanced maternal age and maternal and neonatal morbidity: A cross-sectional study on a Spanish population Mastrolia SA editor. PLoS ONE.

[19] Oliveira FC, SuritaFG,Pinto E Silva JL, Cecatti JG, Parpinelli MA, Haddad SM,  (2014). Severe maternal morbidity and maternal near miss in the extremes of reproductive age: results from a national cross- sectional multicenter study. BMC Pregnancy Childbirth.

[20] Shrim A, Levin I, Mallozzi A, Brown R, Salama K, Gamzu R, et al. Does very advanced maternal age, with or without egg donation, really increase obstetric risk in a large tertiary center? Journal of Perinatal Medicine. 2010 Jan 1;38(6). Available from: https://www.degruyter.com/document/doi/10.1515/jpm.2010.084/html [cited 2023 Nov 25] 10.1515/jpm.2010.084 20707613

[21] Smithson SD, Greene NH, Esakoff TF (2022). Pregnancy outcomes in very advanced maternal age women. American Journal of Obstetrics & Gynecology MFM.

[22] Tunçalp Ö, Souza J, Hindin M, Santos C, Oliveira T, Vogel J (2014). Education and severe maternal outcomes in developing countries: a multicountry cross-sectional survey. BJOG.

[23] Sheen JJ, Wright JD, Goffman D, Kern-Goldberger AR, Booker W, Siddiq Z (2018). Maternal age and risk for adverse outcomes. Am J Obstet Gynecol.

[24] Blankenship SA, Raghuraman N, Delhi A, Woolfolk CL, Wang Y, Macones GA (2020). Association of abnormal first stage of labor duration and maternal and neonatal morbidity. Am J Obstet Gynecol.

[25] Bramham K, Parnell B, Nelson-Piercy C, Seed PT, Poston L, Chappell LC (2014). Chronic hypertension and pregnancy outcomes: systematic review and meta-analysis. BMJ.

[26] Panaitescu AM, Syngelaki A, Prodan N, Akolekar R, Nicolaides KH (2017). Chronic hypertension and adverse pregnancy outcome: a cohort study. Ultrasound in Obstet & Gyne.

